# Tibiofemoral joint contact forces increase with load magnitude and walking speed but remain almost unchanged with different types of carried load

**DOI:** 10.1371/journal.pone.0206859

**Published:** 2018-11-05

**Authors:** Gavin K. Lenton, Peter J. Bishop, David J. Saxby, Tim L. A. Doyle, Claudio Pizzolato, Daniel Billing, David G. Lloyd

**Affiliations:** 1 Gold Coast Orthopaedics Research, Engineering and Education Alliance, Menzies Health Institute Queensland, School of Allied Health Sciences, Griffith University, Southport, Queensland, Australia; 2 Department of Health Professions, Faculty of Medicine and Health Sciences, Macquarie University, New South Wales, Australia; 3 Land Division, Defence Science and Technology Group, Fishermans Bend, VIC, Australia; The Ohio State University, UNITED STATES

## Abstract

Musculoskeletal injuries (MSI) in the military reduce soldier capability and impose substantial costs. Characterizing biomechanical surrogates of MSI during commonly performed military tasks (e.g., load carriage) is necessary for evaluating the effectiveness of possible interventions to reduce MSI risk. This study determined the effects of body-borne load distribution, load magnitude, and walking speed on tibiofemoral contact forces. Twenty-one Australian Army Reserve soldiers completed a treadmill walking protocol in an unloaded condition and wearing four armor types (standard-issue and three prototypes) with two load configurations (15 and 30 kg) for a total of 8 armor x load ensembles. In each ensemble, participants completed a 5-minute warm-up, and then walked for 10 minutes at both moderate (1.53 m**⋅**s^-1^) and fast (1.81 m**⋅**s^-1^) speeds. During treadmill walking, three-dimensional kinematics, ground reaction forces, and muscle activity from nine lower-limb muscles were collected in the final minute of each speed. These data were used as inputs into a neuromusculoskeletal model, which estimated medial, lateral and total tibiofemoral contact forces. Repeated measures analyses of variance revealed no differences for any variables between armor types, but peak medial compartment contact forces increased when progressing from moderate to fast walking and with increased load (p<0.001). Acute exposure to load carriage increased estimated tibiofemoral contact forces 10.1 and 19.9% with 15 and 30kg of carried load, respectively, compared to unloaded walking. These results suggest that soldiers carrying loads in excess of 15 kg for prolonged periods could be at greater risk of knee MSI than those with less exposure.

## Introduction

Lower-limb musculoskeletal injuries (MSI) reduce soldier capability and impose substantial costs on the person and military, i.e., rehabilitation and salary compensation [1]. Knee injuries are particularly problematic, causing not only short-term impairment but often resulting in chronic degenerative diseases, such as knee osteoarthritis, requiring lifelong remuneration [2, 3]. The increase of knee osteoarthritis incidence with age [2] and years of military service [3] suggests continued exposure to the rigors of military duties leads to serious and chronic MSI. Research should therefore focus on identifying mechanisms of lower-limb MSI to inform injury prevention programs.

In-vitro and in-vivo studies have shown many lower-limb MSIs manifest from structural fatigue of supporting and articular tissues induced by repetitive loading [4, 5]. The repetitive loading results in stress concentrations which are influenced by the complex interaction of anthropometric factors, distribution of external (e.g., ground reaction) and internal (e.g., muscle) forces acting about the site, as well as the state of the tissue (i.e., morphology and material properties) [6]. For example, patellofemoral pain can result from deterioration of cartilage under the patella following intense periods of physical activity [7], while high medial compartment knee loading has been linked to increased medial compartment knee osteoarthritis progression in patients already with the disease [8, 9]. Characterizing internal loads related to the development of MSI during commonly performed military tasks (e.g., load carriage) may help identify the mechanisms of common lower-limb MSI.

Biomechanical modelling enables non-invasive characterization of external and internal biomechanical loads acting about joints, bones, and soft tissues. External biomechanical measures, such as joint kinematics and kinetics, have been reported in experimental studies of heavy load carriage during walking [10, 11], running [12], and other military tasks [13]. However, these measures do not represent musculoskeletal tissue loads (e.g., articular contact forces and bone stresses/strains), which directly influence how tissues respond to physical training, and may more closely relate to certain MSI mechanisms [14]. Recently, Ramsay and colleagues [15] showed tibiofemoral contact forces increased in soldiers during an accelerative task where external load magnitude was increased. From the limited data available in soldiers, it could be inferred that contact force estimates are mostly affected by the external load magnitude. However, no studies have determined whether changing load distribution affects tibiofemoral contact forces. Load-distribution systems which shift body-borne load from the shoulders to the hips via a hip belt have been shown to reduce trunk flexion and rotation [16], and upper back forces [17], but the effects on internal knee loading remain unknown.

The purpose of this study was to determine the effects of body-borne load distribution, external load magnitude, and walking speed on tibiofemoral contact forces using an electromyography-informed neuromusculoskeletal model. We hypothesized that peak tibiofemoral contact forces will increase in response to increasing carried load magnitude and walking speed. Additionally, because joint loading is dictated mostly by task demands, we hypothesized that contact forces will not change between standard-issue body armor and armor bearing some load on the hips.

## Methods

Twenty-one male Australian Army Reserve soldiers (Mean±SD, age: 29.5±7.1 years, height: 1.77±0.08 m, mass 82.8±12.1 kg) participated in this study. Only males were recruited because the female population within Australian Defence is low, and it was impossible to recruit sufficient numbers on which to draw robust scientific conclusions from the data. Participants had no recent (<6 months) or long-term history of lower-limb injury that impaired normal walking. Participants were briefed on the study protocol and provided their written informed consent to participation. Approval for this research was provided by the Departments of Defence and Veterans’ Affairs Human Research Ethics Committee (Protocol 756–14).

Participants completed a standardized treadmill walking protocol in an unloaded condition and four body armor conditions. For the unloaded condition, participants wore an athletic shirt and shorts, and military boots. The four body armor conditions included standard issue Tiered Body Armor System (TBAS) and three armor conditions which incorporated a hip belt (cARM1, cARM2, and pARM1). Each armor condition was configured with 15 kg and 30 kg of external load. Full descriptions and illustrations of the armor types and external load conditions are available in the supplementary material (S1 Table). The unloaded condition was always completed first to allow treadmill walking familiarization, whereas the order of the remaining conditions was randomised and counterbalanced to prevent order effects.

Three-dimensional (3D) full-body kinematics, ground reaction forces and surface electromyography (EMG) from nine lower-limb muscles were synchronously acquired using an 11-camera motion capture system (Vicon Motion Systems Ltd, Oxford, UK) (100 Hz), a fore-aft split belt instrumented treadmill (Advanced Mechanical Technology Inc, USA) (1000 Hz), and a 16-channel wireless telemetry system (Telemyo 900, Noraxon, Arizona, USA) (1000 Hz), respectively. Participants were instrumented with spherical (14 mm diameter), retroreflective markers and marker clusters placed on arms, torso, pelvis, thighs, lower legs and feet [18]. Bipolar dual surface electrodes (Ag/AgCl, Noraxon, Arizona, USA) were placed over 9 lower-limb muscle bellies: medial and lateral gastrocnemii, tibialis anterior, peroneus longus, vastus medialis, vastus lateralis, rectus femoris, biceps femoris, and semitendinosus. Before placement, electrode locations on each participant’s dominant leg were palpated, shaved, lightly abraded, and cleaned with an alcohol swab. A reference electrode was placed over the head of the fibula.

For each condition, each participant first undertook a static kinematic calibration trial followed by treadmill walking trial. Each walking trial consisted of 5 minute warm-up, and then 10 minutes at both moderate (1.53m**⋅**s^-1^) and fast (1.81m**⋅**s^-1^) walking speeds. The two walking speeds represented administrative and approach marching speeds in the Australian Army, respectively. During treadmill walking, 3D whole-body kinematics, ground reaction forces and EMG were collected for 30 seconds in the final minute of each speed. Following a minimum of 25 minutes rest (range 25–40 minutes), participants completed the same treadmill walking protocol for the remaining conditions they were assigned in the testing session. To complete the nine total experimental conditions, participants completed three different armor conditions (i.e., armor type and load) per testing session across three separate testing days. Only three experimental conditions were completed per session to reduce the risk of muscular fatigue developing within the session. Subsequent testing sessions were separated by at least two days to minimize fatigue effects.

Marker trajectories from static calibration and walking trials were reconstructed and marker gaps (<10 frames) interpolated using cubic splines in Vicon Nexus version 2.5. A modified version of MOtoNMS [19], a Matlab (R2016b, The Mathworks) toolbox for biomechanics data processing, was used to process marker, force plate, and EMG data for use in OpenSim [20]. In this toolbox, the hip joint center was defined using Harrington regression [21], and knee and ankle joint centers defined using midpoints of medial and lateral femoral condyles and malleoli, respectively. Right heel-strike events in treadmill walking trials were determined from the distance between right calcaneus and sacrum markers [22], and subsequently used to crop 30 s data acquisitions into complete gait cycles. Marker trajectories and ground reaction forces were low-pass filtered (6 Hz) using a 2^nd^ order Butterworth design [23], and transformed from the laboratory to the OpenSim coordinate system. Raw EMG data were corrected for direct current offset, band-pass filtered (30-450Hz), full-wave rectified, and low pass filtered (6Hz) to yield linear envelopes. Linear envelopes were then amplitude normalized to peak EMG values calculated from all treadmill walking trials within each session to ensure values were below one [24].

Kinematics and kinetics were determined using OpenSim version 3.3 [20]. A generic full-body OpenSim musculoskeletal model with 40 muscle-tendon units per lower-limb was used [25], which included three rotational degrees of freedom (DOF) for the hip, one DOF for the knee, with abduction/adduction and internal/external rotations prescribed as a function of knee flexion angle, and one DOF for the ankle. Modifications were made to the original model to allow computation of 3D knee moments and tibiofemoral contact forces. Briefly, an additional and massless ‘dummy’ tibia body was first added to the model as a child body of the original tibia. A custom joint connecting the tibia and its dummy was defined such that the dummy tibia and actual tibia motions were coincident, and motion between bodies was locked. Since there were 6 generalized coordinates (i.e., DOFs) parameterizing this joint, sagittal-, frontal- and transverse-plane knee joint moments could be solved for. Additionally, two contact points were added to the knee to allow computation of net joint moments and muscle tendon unit moment arms about the tibia-fixed medial and lateral compartments [26]. The distance separating the two points on the mediolateral axis was individualized for each participant’s anatomy based on their femoral condyle width, which was defined from markers on the femoral condyles [27].

The generic OpenSim musculoskeletal model was linearly scaled to each participant’s anthropometry. An additional optimization procedure was used to preserve dimensionless optimal fibre and tendon slack length operating ranges for each muscle-tendon actuator [28]. This model was subsequently used in inverse kinematics [29] and inverse dynamics (ID) tools to determine each participant’s and lower-limb joint kinematics and net external moments, respectively. The OpenSim muscle analysis tool was used to obtain muscle-tendon unit (MTU) lengths and moment arms with respect to all lower-limb joint DOFs.

Following this, the calibrated, EMG-informed neuromusculoskeletal modelling (CEINMS) OpenSim toolbox [30] was used to estimate MTU forces and tibiofemoral contact forces about medial and lateral contact points. To-do-so, CEINMS used as inputs external lower-limb joint moments, EMG-derived excitations and MTU lengths and moment arms. CEINMS neuromusculoskeletal model parameters that defined muscle activation and contraction dynamics were first calibrated for each participant to produce muscle-driven moments which matched the ID-derived knee flexion-extension moments while simultaneously minimizing tibiofemoral contact forces [27, 31]. We used CEINMS in EMG-assisted mode [30] as described by Sartori and colleagues [32], whereby optimization adjusted EMG-derived excitations and synthesized muscle excitations for which no experimental EMGs were available (e.g., gracilis, sartorius, and tensor fasciae latae), to ensure close tracking of all lower-limb joint moments and EMG-derived excitations, while minimizing the sum of squared excitations. Estimated muscle forces were then used to determine medial and lateral compartment tibiofemoral contact forces using the planar knee mechanism described above [26]. All contact force estimates were scaled to each participant’s bodyweight (BW).

The contribution of muscle and external (i.e., knee adduction moment) loads to tibiofemoral contact force about each compartment [27] was calculated to examine if any of the experimental conditions affected how frontal plane external loads were stabilized. We calculated these contributions by first summing external knee adduction and frontal plane contact moments (total external), and muscle moments about both the medial and lateral contact points. Total external and muscle contributions were then determined by calculating their relative contribution to total compartment loading.

**Fig 1:**
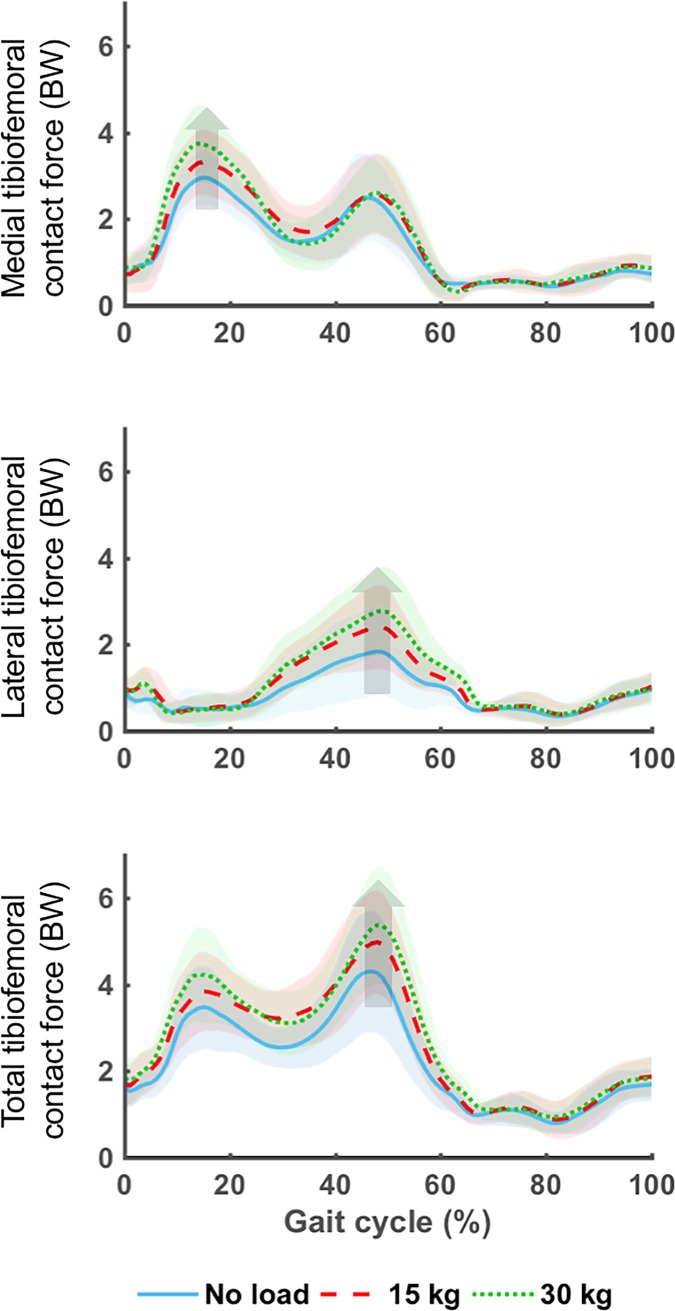
Mean (lines) and standard deviation (shaded region) medial, lateral, and total tibiofemoral contact forces for the unloaded, 15 kg, and 30 kg load conditions normalized to a gait cycle. Data for all loads were aggregated for walking speed. Arrows indicate significant increases in peak contact force with increasing load.

**Fig 2:**
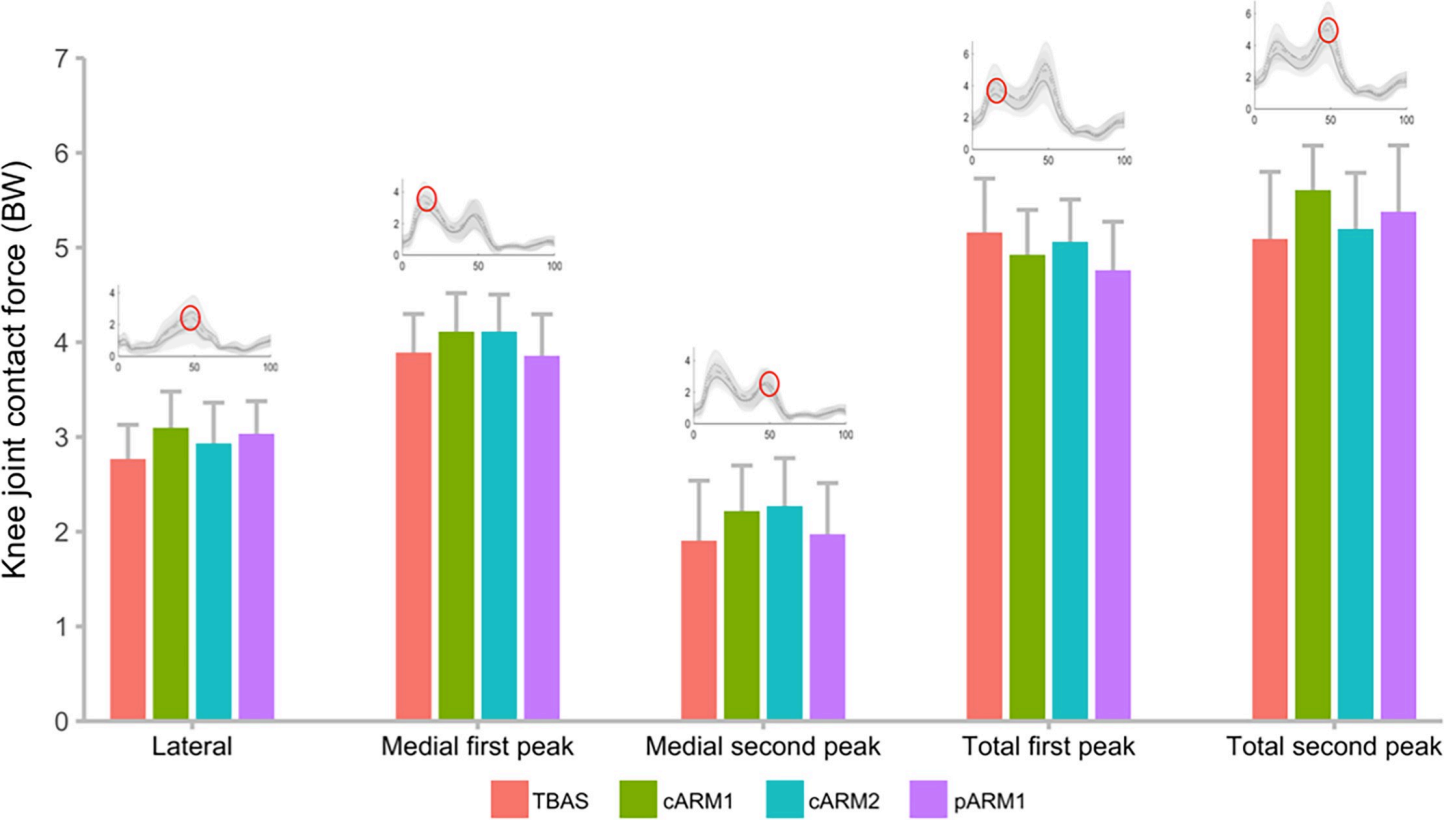
Mean ± 95% confidence intervals of peak tibiofemoral contact forces normalized to body weight (BW) and compared across the different armor types during fast walking while carrying 30 kg of load.

Each participant’s walking trials were cropped and normalized to 100% of the gait cycle (initial foot contact to next ipsilateral initial foot contact) and ensemble-averaged to generate mean waveforms for every condition. From this data, the two main peaks of medial compartment, first peak of lateral compartment, and first two peaks of total (i.e., sum of medial and lateral) tibiofemoral contact forces (Figs 1 and 2) were extracted for subsequent statistical analyses in R version 3.3.1 (RStudio, Inc, Boston, MA) v0.99.903. Shapiro-Wilk tests confirmed normally distributed data, and to allow comparisons between all load conditions, separate two-way (load x speed) repeated measures Analyses of Variance (ANOVA) were performed using data from the unloaded condition and the 15 kg and 30 kg conditions obtained from TBAS. Additionally, three-way repeated measures ANOVAs were used to test for significant interactions between, and main effects of, armor type, external load condition and/or walking speed for peak tibiofemoral contact forces. Post-hoc paired t-tests were performed for significant interactions or main effects to identify specific differences, with p-values adjusted as per Benjamini and Hockberg [33] to control for multiple comparisons. Significance was set at p<0.05. Partial eta-squared (η^2^p) effect sizes were calculated with small, medium, and large effects defined as η^2^p between 0.01 and 0.06, 0.06 and 0.14, and greater than 0.14, respectively [34].

## Results

There were no significant interactions in the two-way ANOVA between walking speed and carried load for any peak joint contact forces. There were, however, significant main effects for walking speed for the first peak of medial (F = 11.26, p = 0.001) and total (F = 9.59, p = 0.002) knee JCF. Specifically, fast walking compared to moderate walking speeds elicited higher first peaks of the medial (3.61±0.78 BW versus 3.15±0.78 BW) and total (4.33±1.10 BW versus 3.75±0.96BW) knee JCF (p<0.05). There were also significant main effects of external load for first peak of medial (F = 6.55, p = 0.002), lateral (F = 4.89, p = 0.009), and second peak of total (F = 6.64, p = 0.002) knee JCF (Fig 1). Carrying 15 kg and 30 kg of load resulted in 10.1% and 19.9% increases in peak medial knee JCF compared to values obtained when walking without load, respectively. Additionally, when carrying 30 kg, second peak of total knee JCF was 28.3% higher compared to carrying unloaded, and 12.9% higher compared to values obtained when carrying 15 kg.

**Fig 3:**
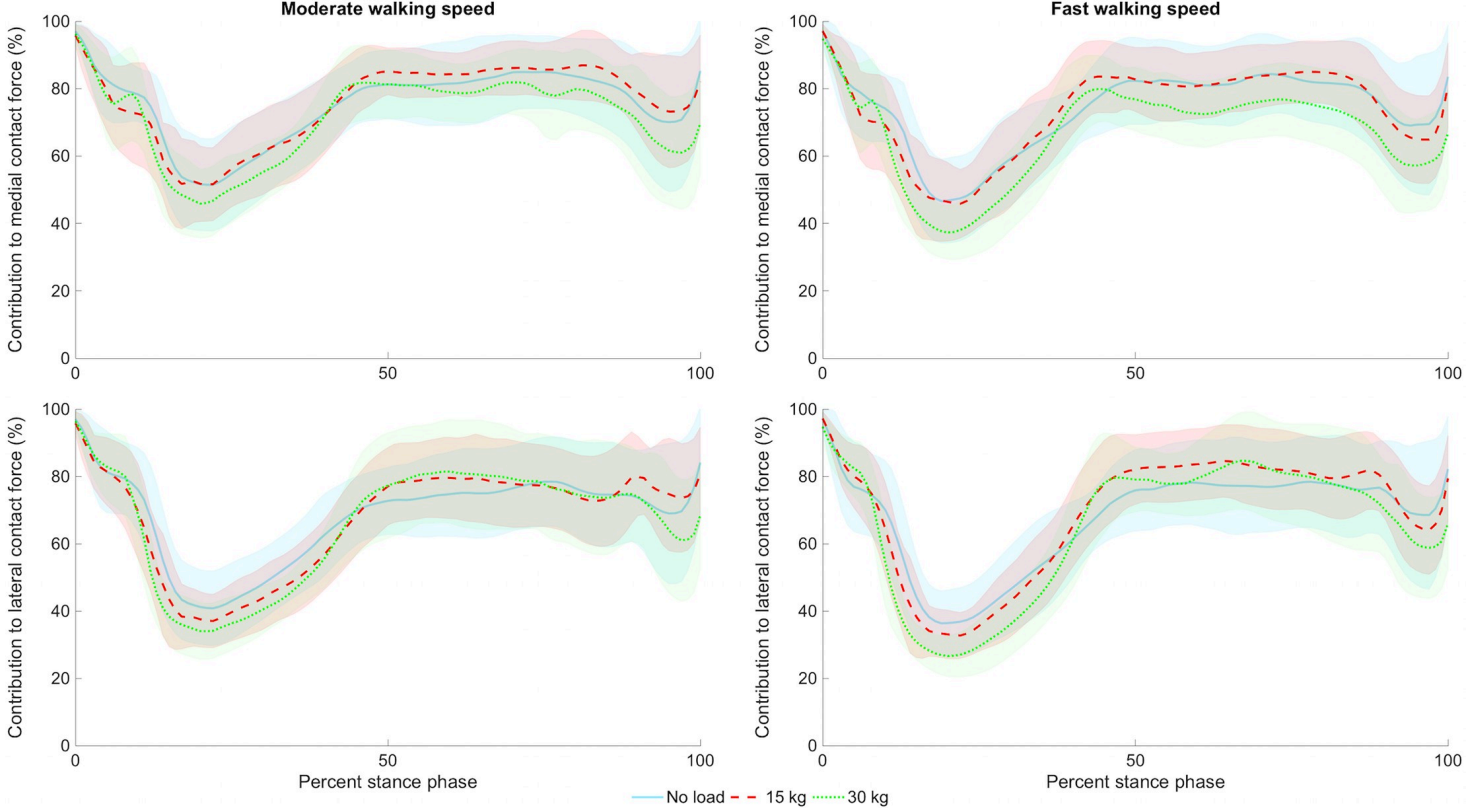
Mean ± SD contribution of muscles to medial (top row) and lateral (bottom row) compartment tibiofemoral contact force for the no load (solid light blue line), 15 kg (dashed red line), and 30 kg (dotted green line) conditions during moderate (first column) and fast (second column) walking.

Three-way repeated measures ANOVAs revealed no interactions between body armor type, walking speed, and/or carried load for any peak contact force variable (Table 1). There were no main effects of armor type for any variable and effect sizes were small. Even in the most physically demanding configuration (i.e., fast walking and carrying 30 kg), no differences were observed between armor types (Fig 2), with first peak of medial contact force reaching ~4 times body weight regardless of the armor type. Additionally, there were no interactions or main effects of armor type, walking speed, and carried load for muscle and external load contributions to tibiofemoral contact force (Table 2, Fig 3). Regardless of the armor type, muscles contributed >65 and >70% to lateral and medial compartment contact forces, respectively. Moreover, approximately 20% of the contact loading was attributed to the quadriceps muscles. Thus, while the magnitude of tibiofemoral contact forces increase with increases in carried load and walking speed, relative contributions of muscle and external load to these forces did not change.

**Table 1:** Main effects of armor type for medial, lateral, and total tibiofemoral contact forces normalized to body weight (BW).

N = 80	Armor type						
	TBAS	cARM1	cARM2	pARM1	F	p-value	η^2^p^a^
**Medial first peak**	3.62±0.91	3.69±0.85	3.57±0.87	3.51±0.74	0.74	0.529	0.007
**Medial second peak**	2.37±1.04	2.34±1.17	2.13±1.05	2.19±1.22	0.87	0.456	0.009
**Lateral peak**	2.77±0.84	2.87±0.81	2.91±0.82	2.67±0.84	1.33	0.264	0.013
**Total first peak**	4.44±1.21	4.57±1.18	4.57±1.12	4.41±1.06	0.47	0.703	0.005
**Total second peak**	4.88±1.34	5.01±1.37	4.86±1.45	4.82±1.38	0.29	0.830	0.003

^a^η^2^p, partial eta squared.

**Table 2:** Mean ± SD percent contribution (%) of all knee-spanning muscles, external load, and quadriceps muscles to medial and lateral compartment tibiofemoral contact force in the stance phase of gait. All data values have been aggregated for the different load and speed conditions.

N = 80	Armor type					
	TBAS	cARM1	cARM2	pARM	p-value	η^2^p^a^
Medial muscle contribution	71.4 ± 7.4	70.9 ± 6.6	72.9 ± 7.3	71.9 ± 5.9	0.251	0.013
Medial vastii contribution	19.6 ± 6.4	19.7 ± 6.9	20.0 ± 7.0	20.4 ± 7.0	0.898	0.002
Medial external contribution	28.5 ± 7.6	29.0 ± 6.3	27.0 ± 7.3	28.2 ± 5.9	0.293	0.012
Lateral muscle contribution	66.7 ± 7.1	67.7 ± 6.5	66.9 ± 6.7	67.7 ± 7.6	0.693	0.005
Lateral vastii contribution	20.4 ± 6.3	20.8 ± 6.5	21.8 ± 7.1	21.1 ± 7.2	0.579	0.006
Lateral external contribution	33.0 ± 6.9	32.6 ± 6.5	33.2 ± 7.0	32.3 ± 7.4	0.836	0.003

^a^η^2^p, partial eta squared.

## Discussion

This study assessed the effects of different body-borne load distributions, load magnitudes, and walking speeds on tibiofemoral contact forces in soldiers while they walked. We found that the type of body armor ensemble had no influence on peak tibiofemoral contact forces, regardless of carried load or walking speed. The first peak of medial compartment contact force and second peak of total contact force increased in response to increasing load magnitude and walking speed. During fast walking while carrying 30 kg of load, peak total tibiofemoral contact forces were ~5 times body weight. To our knowledge, this is the first study to explore tibiofemoral contact forces during walking with different carried loads and walking speeds in a soldier population.

Agreeing with the first hypothesis, tibiofemoral contact forces did not change when soldiers wore body armor incorporating a hip belt compared to armor without a hip belt (Table 1). This was confirmed by non-significant ANOVA interactions and main effects, and small effect sizes for all comparisons. Taken together with studies assessing changes in external biomechanics (e.g., torso angles and torso/pelvis coordination) [16, 17], it appears that altering armor load distribution with hip belts minimally affects biomechanical surrogates of MSI at the knee and torso during gait. Given no other study has reported changes in tibiofemoral contact forces due to armor type or load distributions (e.g., high vs. low load placement in backpacks), we cannot directly compare our results with other literature values. However, studies have shown slight variations in lower-limb joint kinematics between different load placements [10, 35], although altered kinematics is not necessarily indicative of altered internal joint biomechanics, because muscle forces and external loads could be different. In the current study, muscle and external load contributions to total tibiofemoral contact force were the same between armor types, loads, and walking speeds (Table 2, S3 Table). Thus, the kinematics, ground reaction forces, and muscle forces changed in a way that led to similar tibiofemoral contact forces.

The absence of change between armor types suggests exposure to different task demands (e.g., load magnitude, walking speed, terrain, and gradient) influences biomechanical surrogates of MSI risk more than body-borne load distribution. However, we only tested three load sharing designs for two armor load configurations during steady-state, level walking. Thus, testing novel designs across a broad range of physical tasks and environmental scenarios is necessary to extend the generalizability of results.

Consistent with our second hypothesis, and a prior study of run-to-stop maneuvers in soldiers [15] and simulated increases in body weight [36], tibiofemoral contact forces increased in response to greater carried load. Similarly, increasing walking speed increased the first peak of medial compartment tibiofemoral contact forces. These results agree with previous research demonstrating tibiofemoral contact forces during weight acceptance are sensitive to changes in walking speed [37]. The current study suggests that compared to unloaded walking at moderate speed, carrying load at a fast speed causes increased external loads following heel strike that muscle forces must counteract to prevent the lower-limb joints from collapsing, and thereby increase tibiofemoral contact forces. Importantly, these forces approximate those observed during running (~6 x BW) [27, 37], and may place soldiers at higher than normal risk for lower-limb MSI. Continued exposure to high contact forces may explain why soldiers are susceptible to degenerative joint diseases during and following military service [2, 3]. Individuals walking with high knee adduction moments, which, along with EMG amplitude, is a modest surrogate of in-vivo knee loading [38, 39], have been shown to have faster progression to late stage knee osteoarthritis compared to those with lower adduction moments [8]. However, knee adduction moment is a less convincing predictor of injury initiation [40]. Therefore, additional research should be explored to identify if the contact forces estimated in this study lead to the initiation or progression of knee osteoarthritis.

**Fig 4:**
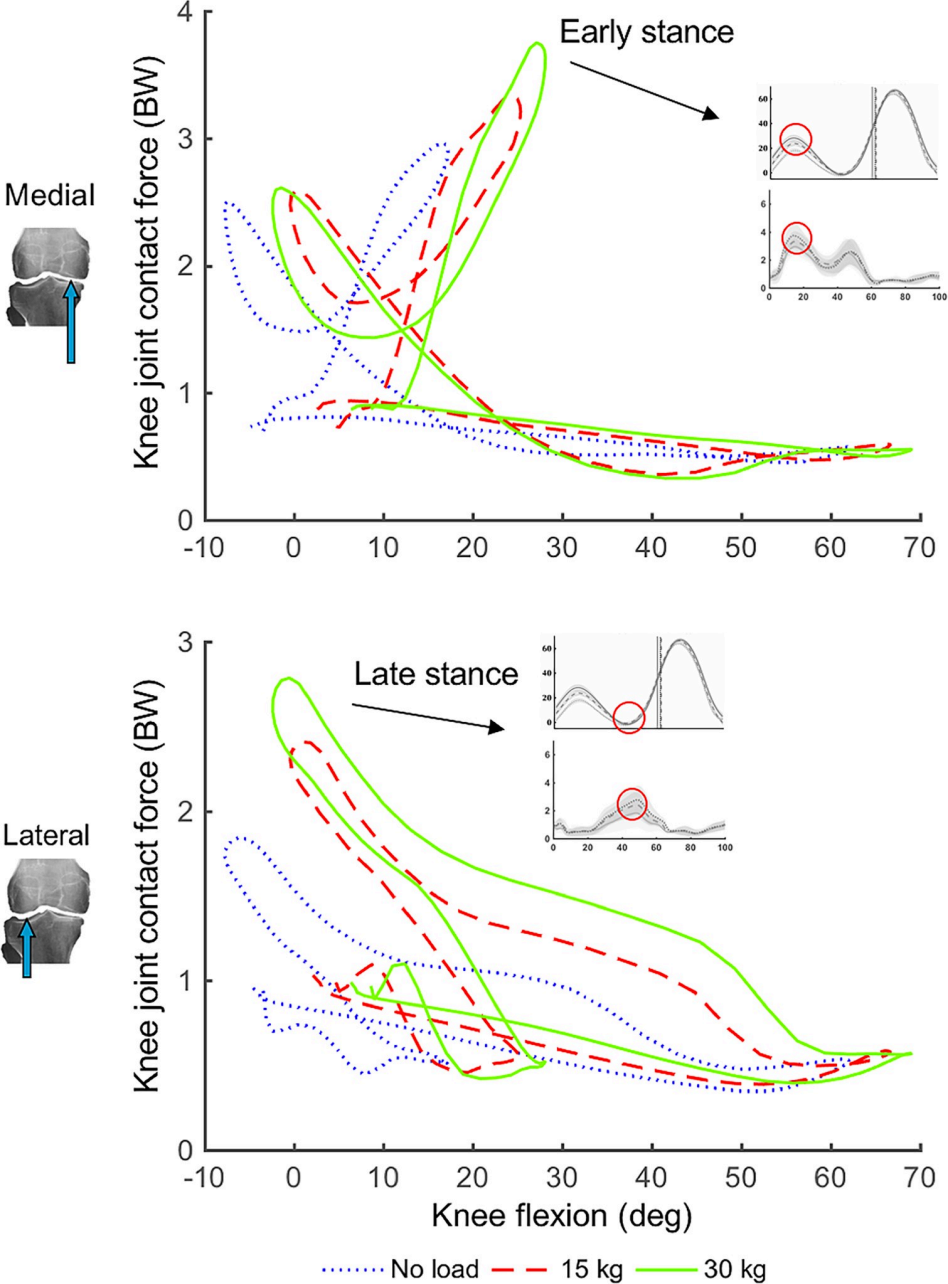
Plots of knee flexion angle versus medial (top) and lateral (bottom) compartment tibiofemoral contact forces across the different load conditions. Both plots have been annotated with time-series plots of knee flexion angle and corresponding tibiofemoral contact forces with peak contact force timing circled red.

Future interventions to reduce MSIs could focus on reducing knee contact forces during load carriage by designing assistive devices to transfer or absorb the load. Temporally aligning device assistance to provide the best user benefits has proven difficult in previous experiments of unloaded walking, whereby assistance timing is manually modified or fine-tuned to each user [41, 42]. As an alternative, our plots of medial and lateral contact force versus knee flexion angle (Fig 4) suggest knee flexion rather than gait cycle timing could inform knee assistive device timing during load carriage. This is because peak knee flexion angle in early stance consistently coincided with the first peak of medial compartment contact force across different carried load conditions. Given researchers can easily and reliably measure knee flexion angle using motion capture or inertial measurement units, it could be a robust input parameter for device control (e.g., setting knee flexion as threshold for assistance).

This study has some experimental and modelling limitations that should be acknowledged. First, although experimental EMGs were used to estimate MTU forces of major muscles surrounding the knee, there were model MTUs which had excitations synthesized from static optimization (e.g., sartorius, gracilis). We were limited to nine experimental EMG sites and, therefore, selected the primary muscles spanning the knee. However, EMG-informed methods provide good estimates of measured tibiofemoral contact forces compared with in-vivo measured data [26, 27, 31]. Second, to not expose participants to a sudden increase in walking speed, we always prescribed moderate walking before fast. This means comparisons between walking speeds must be interpreted with caution, as order effects may be present. Third, we used generically-scaled musculoskeletal models for contact force estimates and not subject-specific models built from medical imaging. Knee joint geometry, MTU lengths, and MTU moment arms may therefore not be completely representative of each participant and this could reduce the accuracy of contact force predictions [31]. However, given that we used within-subject comparisons, any errors in estimations were consistent within each participant and should not have influenced study findings. Fourth, a combination of EMG-driven modelling and static optimization were used to estimate individual muscle forces, which were subsequently used to determine tibiofemoral contact forces. While the EMG-driven approach has been validated against in-vivo data [43], the EMG-driven/static optimization hybrid approach has not, and suffers from the same muscle redundancy problem inherent in an overdetermined system [44]. Moreover, due to no currently available in-vivo data, direct validation of our muscle force estimates is not yet possible in healthy individuals during physically intense tasks (e.g., load carriage walking). Finally, although one study revealed males and females have similar walking mechanics during load carriage [11], only males were tested in this study, thereby limiting applicability of results to females.

In conclusion, this was the first study to estimate tibiofemoral contact forces, in a military population, during walking using an EMG-informed neuromusculoskeletal model. Peak contact forces were similar between armor types and, therefore, load sharing systems appear to minimal influence this potential biomechanical surrogate of knee MSI. Despite no change between armor types, acute exposure to load carriage caused large medial and lateral compartment tibiofemoral contact forces, the magnitude of which increased in response to increased external load and/or walking speed. Regular exposure to contact forces of these magnitudes may precipitate development and progression of knee overuse injuries, although prospective radiographical evidence of disease initiation and progression is required to substantiate this claim. In any case, military organizations should implement strategies to reduce soldier exposure to high magnitude contact forces. Given load carriage will continue to be an unavoidable soldier requirement, we suggest improved physical conditioning and/or the use of assistive devices as ways to mitigate current injury burden. However, further research is required to establish evidence-based designs for either approach.

## Supporting information

S1 Table Descriptions and illustrations of different types of body armor and load configurations. (DOCX)

S2 Table Root mean square error (RMSE) and coefficient of determination (R^2^) for CEINMS-derived torques and inverse dynamics-derived torques during stance phase. (DOCX)

S3 Table Muscle contributions to medial and lateral tibiofemoral contact forces across the different carried loads and walking speeds. (DOCX)

S1 File Stance phase medial compartment tibiofemoral contact force for the no load, 15 kg, and 30 kg load magnitudes. (CSV)

S2 File Stance phase lateral compartment tibiofemoral contact force for the no load, 15 kg, and 30 kg load magnitudes. (CSV)

S3 File Stance phase total tibiofemoral contact force for the no load, 15 kg, and 30 kg load magnitudes. (CSV)

S4 File Peak medial and lateral compartment, and total tibiofemoral contact forces for each armour type and walking speed configuration used for statistical comparisons. (CSV)

S5 File Peak medial and lateral compartment, and total tibiofemoral contact forces for walking speed and load magnitude used for statistical comparisons, including the no load condition. (CSV)
