# Supplementary material for: Tibiofemoral joint contact forces increase with load magnitude and walking speed but remain almost unchanged with different types of carried load
Source: PLoS One. 2018 Nov 5;13(11):e0206859. doi: 10.1371/journal.pone.0206859 (PMC6218072; doi:10.1371/journal.pone.0206859)
Supplement: S1 Table — (DOCX) [file pone.0206859.s001.docx]

S1 Table. Description of the armour vests and load distribution devices (LDD) used for testing.

| Armour | Vest | LDD | Description |
| --- | --- | --- | --- |
| TBAS | 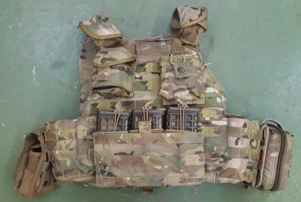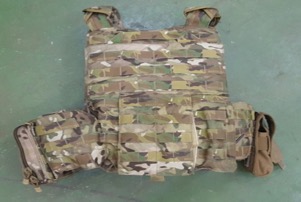 | N/A | No hip belt was included in this armour system |
| cARM1 | 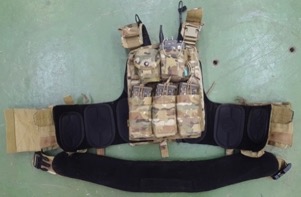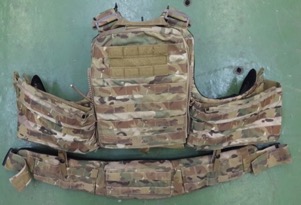 | **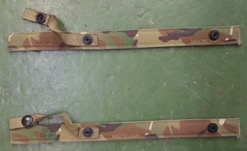** | Armour Chassis with a structural kinetic load sharing system that links the belt to the torso vest using two fibreglass struts positioned above the iliac crest. The struts attach from the base of the vest to the top of the belt via pockets, and strap in place via Modular Lightweight Load-carrying Equipment (MOLLE) loops sitting vertically between belt and vest located on both sides of the system. |
| cARM2 | 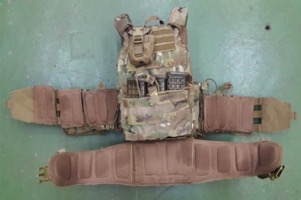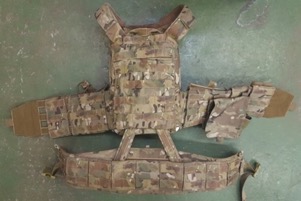 | **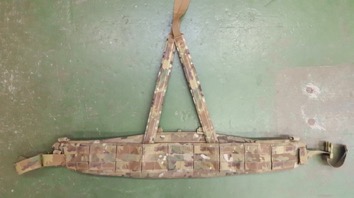** | Uses stays made from polyethylene, nylon, and carbon fibre in an X-formation that connect from the ballistic vest to the Brokos belt. The stays originate at the top and back of the belt, crossing over and inserting into the base and back of the vest. |
| pARM1 | 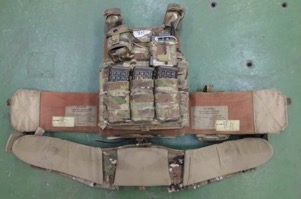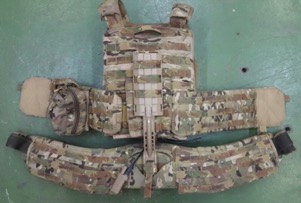 | **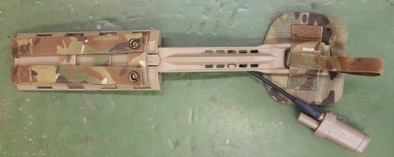** | A polyethylene, curved spine located at the rear of the belt. The spine originates at the top of the armour vest and into a pocket at the rear. The spine length can be adjusted using a button-release sliding mechanism. |

Load configurations

The 15 kg load consisted of an armour system (i.e. torso vest and hip belt) with replica ballistic soft armour inserts and aluminium training plates that replicated the same shape and mass of ballistic plates, three F88 magazine cartridges weighted to represent the shape and mass of a full magazine, a replica grenade and multiband inter/intra team radio fitted to the front of the vest, and a medical pouch and extra magazine cartridge fitted to the left and right side of the torso vest cummerbund, respectively. The location of each pouch was chosen after consultation with military advisors and represented an operational configuration, with all pouches filled with weights of approximate size and mass to the actual field equipment. The 30 kg condition consisted of the same 15 kg of tactical items, plus a medium-sized assault pack that coupled onto the rear of the torso vest. This assault pack was loaded with another 15 kg of mass, evenly distributed throughout the pack to add bulk without concentrating the mass to a specific location in the pack.
