# Supplementary material for: Tibiofemoral joint contact forces increase with load magnitude and walking speed but remain almost unchanged with different types of carried load
Source: PLoS One. 2018 Nov 5;13(11):e0206859. doi: 10.1371/journal.pone.0206859 (PMC6218072; doi:10.1371/journal.pone.0206859)
Supplement: S2 Table — (DOCX) [file pone.0206859.s002.docx]

S2 Table. Root mean square error (RMSE) and coefficient of determination (R^2^) for CEINMS-derived torques and inverse dynamics-derived torques during stance phase

| Condition | RMSE (Nm/kg) | R2 |
| --- | --- | --- |
| No armour – moderate | 0.12 ± 0.05 | 0.62 ± 0.12 |
| No armour – fast | 0.16 ± 0.06 | 0.69 ± 0.13 |
| 15 kg – moderate | 0.15 ± 0.05 | 0.57 ± 0.15 |
| 15 kg – fast | 0.19 ± 0.08 | 0.56 ± 0.15 |
| 30 kg – moderate | 0.18 ± 0.07 | 0.62 ± 0.11 |
| 30 kg – fast | 0.26 ± 0.11 | 0.47 ± 0.18 |
