# Supplementary material for: Tibiofemoral joint contact forces increase with load magnitude and walking speed but remain almost unchanged with different types of carried load
Source: PLoS One. 2018 Nov 5;13(11):e0206859. doi: 10.1371/journal.pone.0206859 (PMC6218072; doi:10.1371/journal.pone.0206859)
Supplement: S3 Table — (DOCX) [file pone.0206859.s003.docx]

S3 Table. Mean ± SD percent contribution (%) of all knee-spanning muscles, external load, and quadriceps muscles to medial and lateral compartment tibiofemoral contact force in the stance phase of gait. All data values have been aggregated for armour types. ^a^ indicates significant differences in absolute magnitude of the variable between walking speeds, ^b^ indicates significant differences in absolute magnitude of the variable between loads, ^c^ indicates significant interaction between speed and load.

|  | Walking Speed | | η^2^p^a^ | Load | | η^2^p | Speed/Load | η^2^p |
| --- | --- | --- | --- | --- | --- | --- | --- | --- |
|  | Moderate | Fast |  | 15 kg | 30 kg |  | p-value |  |
| Medial muscle contribution^a,b^ | 73.1 ± 6.8 | 70.4 ± 6.6 | 0.041 | 72.7 ± 6.4 | 70.8 ± 7.1 | 0.022 | 0.908 | 0.000 |
| Medial vastii contribution | 19.8 ± 6.9 | 20.0 ± 6.7 | 0.000 | 20.0 ± 7.0 | 19.8 ± 6.6 | 0.000 | 0.699 | 0.000 |
| Medial external contribution^a,b^ | 26.9 ± 6.7 | 29.4 ± 6.7 | 0.036 | 27.2 ± 6.3 | 29.1 ± 7.2 | 0.022 | 0.895 | 0.000 |
| Lateral muscle contribution | 67.3 ± 7.3 | 67.3 ± 6.7 | 0.000 | 67.8 ± 6.8 | 66.7 ± 7.2 | 0.006 | 0.930 | 0.000 |
| Lateral vastii contribution | 20.9 ± 6.8 | 21.1 ± 6.8 | 0.000 | 20.9 ± 7.0 | 21.2 ± 6.5 | 0.000 | 0.980 | 0.000 |
| Lateral external contribution | 32.8 ± 7.2 | 32.7 ± 6.6 | 0.000 | 32.1 ± 6.7 | 33.4 ± 7.1 | 0.008 | 0.901 | 0.000 |
